# Social-Aware Peer Discovery for Energy Harvesting-Based Device-To-Device Communications

**DOI:** 10.3390/s19102304

**Published:** 2019-05-18

**Authors:** Zelalem Legese Hailemariam, Yuan-Cheng Lai, Yen-Hung Chen, Yu-Hsueh Wu, Arthur Chang

**Affiliations:** 1 Department of Information Management, National Taiwan University of Science and Technology, Taipei 106, Taiwan; zolacool2@gmail.com (Z.L.H.); laiyc@cs.ntust.edu.tw (Y.-C.L.); a0926622943@gmail.com (Y.-H.W.); 2 Department of Information Management, National Taipei University of Nursing and Health Sciences, Taipei 112, Taiwan; 3 Bachelor Program in Interdisciplinary Studies, National Yunlin University of Science and Technology, Yunlin 640, Taiwan; changart@yuntech.edu.tw

**Keywords:** Device-to-Device (D2D), peer discovery, energy harvesting, social awareness

## Abstract

In Device-to-Device (D2D) communications, the first step is to find all of the neighboring peers in the network by performing a peer discovery process. Most previous studies use the social behaviors of the users to adjust the sending rates of the peer discovery messages (i.e., beacons) under the constraint of consumed power for increasing the Peer Discovery Ratio (PDR). However, these studies do not consider the potential for energy harvesting, which allows for the User Equipments (UEs) to procure additional power within charging areas. Accordingly, this paper proposes an Energy-Ratio Rate Decision (ERRD) algorithm that comprises three steps, namely Social Ratio Allocation (SRA), Energy Ratio Allocation (ERA), and Beacon Rate Decision (BRD). The SRA step determines the allocated power quantum for each UE from the total budget power based on the social behavior of the UE. The ERA step then adjusts this allocated power quantum in accordance with the power that is harvested by the UE. Finally, the BRD step computes the beacon rate for the UE based on the adjusted power quantum. The simulation results show that ERRD outperforms the previously-reported Social-Based Grouping (SBG) algorithm by 190% on the PDR for a budget power of one watt and 8% for a budget power of 20 watts.

## 1. Introduction

The demands that are placed on wireless communications have exponentially increased in recent years due to the proliferation of User Equipments (UEs) and the unceasing development of new mobile services. Consequently, the lack of spectrum resources has emerged as a significant concern for communication operators. Currently, any UE that wishes to transmit data to another UE must transmit this data via the Base Station (BS) in the cellular network. However, if the distance between the two UEs is sufficiently small, then the potential exists for the UEs to communicate directly, thereby saving cellular spectrum resources. Accordingly, a new communication paradigm designated as Device-to-Device (D2D) communication has been proposed as a means of achieving short-distance transmissions in 5G networks with improved resource utilization efficiency.

In implementing D2D communications, the first, and most critical, step is that of peer discovery, in which the UEs attempt to identify all of their neighbors in the network [1]. The peer discovery process is generally performed using a beacon mechanism and it aims to maximize the Peer Discovery Ratio (PDR), which is defined as the number of peers that are found in the discovery process divided by the total number of peers in the network. Most previous studies on peer discovery focus on the problems of improving the efficiency of the search process, minimizing the number of collisions, and determining a suitable beacon rate for the UEs [2,3,4,5,6,7,8,9,10,11,12,13,14]. 

In practice, a large proportion of D2D communications stems from the interaction between users over social media, such as Facebook, Twitter, Plurk, and so on [15,16]. Consequently, many studies have investigated the problem of social-aware peer discovery [17,18,19,20,21]. In general, the results have shown that the social behavior of the users provides a useful tool for adjusting the beacon rates of the UEs in such a way to improve the overall efficiency of the peer discovery process [17,18,19,20,21].

Energy (Energy and power are used interchangeably in this paper) is a critical concern in D2D communications, since the UEs generally only have limited energy resources and they often consume energy extremely rapidly when running user applications. Furthermore, the devices (e.g., mobile phones/sensors) in mine pine or oil well also encounter the energy issue, because they cannot recharge the power with any metal contact, hence avoiding the sparkles and reducing the explosion probabilities. Energy harvesting has thus attracted growing interest in recent years as a means of enabling UEs to scavenge energy from surrounding energy sources, especially for limited capacity energy storage electrical devices and systems [22,23,24,25,26,27,28,29]. Broadly speaking, the scavenged energy can be classified as either renewable energy or nonrenewable energy. In the former case, the energy is obtained from natural sources (e.g., thermal, solar, and wind), while in the latter case, the energy is obtained from artificial sources (e.g., electromagnetic resonance, electromagnetic induction, and radio frequency). The energy harvesting mainly adopts the technology of wireless power transfer (WPT) via radio frequency (RF) to transmit power, and then uses the rectifying antenna to convert the received radio signal to direct current (DC) and charge the battery [23]. In light of the promising flexibility of energy harvesting, many organizations [24] (i.e., International Telecommunications Union, European Cooperation in Science and Technology, Wireless Power Transfer Consortium for Practical Applications) and scholars [25,26] have been involved in variable application developments, which include drone powered wireless sensor network [27], emergency ubiquitous power source system [28], and wireless power transfer in electric vehicles environments [29]. Some studies have focused on energy harvesting-based D2D networks, which use harvested energy to promote the D2D data communications [30,31,32]. 

As stated above, the literature contains many studies on the use of social behavior mechanisms to enhance the performance of D2D communications. However, while some of these studies actively address the problem of limiting the energy that is consumed in the peer discovery process, none of them consider an environment in which the UEs are able to procure additional energy from the environment while using energy-harvesting techniques. Nonetheless, such a strategy is of considerable benefit in improving the performance of the peer discovery process. In particular, UEs that acquire additional energy can send a greater number of beacon messages (thereby increasing the PDR), while those that acquire no additional energy can reduce their beacon rate in order to minimize the out-of-energy risk and prolong their participation in the discovery process. 

Consequently, the present study proposes a novel peer discovery algorithm for energy-harvesting environments, designated the Energy-Ratio Rate Decision (ERRD) algorithm. ERRD comprises three steps, namely Social Ratio Allocation (SRA), Energy Ratio Allocation (ERA), and Beacon Rate Decision (BRD). SRA determines an initial allocation power quantum for each UE that is based on its social behavior. ERA then adjusts this power quantum based on the harvested power of the UE. Finally, BRD computes the beacon rate for the UE based on the adjusted power quantum. For UEs with a high harvested power, ERRD increases the beacon rate, thereby increasing the PDR. By contrast, for UEs with low (or no) harvested power, ERRD reduces the beacon rate, and hence prolongs the lifetime of the UE, thereby increasing the time for which the UE can perform beacon discovery.

This study attempts to determine the suitable beacon rates of all UEs to maximize PDR for D2D communications in an energy-harvesting environment. The contributions of this paper are across three orientations: (1) regarding the problem orientation, to the best of our knowledge, this study is the first paper to consider the social-aware peer discovery problem for D2D communications in an energy-harvesting environment, (2) regarding the solution orientation, we propose ERRD, which determines the beacon rate according to UE’s social behavior and the harvested power quantum to increase its PDR, and (3) regarding the evaluation orientation, some of the simulations are conducted to investigate many important parameters along with significant observations.

The remainder of this paper is organized, as follows. Section 2 introduces the related work in the field. Section 3 describes the system model that is considered in the present study and formulates the related problem. Section 4 introduces the ERRD algorithm and describes its detailed operation. Section 5 presents and discusses the simulation results. Finally, Section 6 provides some brief concluding remarks and indicates the intended direction of future research.

## 2. Related Works

The problem of peer discovery in D2D communications has attracted considerable attention in the literature. Broadly speaking, existing approaches can be classified as either social-aware or social-unaware (see Table 1). Within each classification, the proposed mechanisms can be further divided as autonomous, network-assisted, or network-controlled. In autonomous mechanisms, the UEs find nearby peers by themselves, and hence the major issue lies in determining efficient methods for broadcasting the beacons with a minimum number of collisions. By contrast, in network-assisted solutions, the BS collects surrounding the information, determines a suitable beacon rate for each UE, and allocates a proper amount of resources to each UE to perform its transmissions. Finally, in network-controlled mechanisms, the BS directly helps the UEs to find peers by locating UEs.

The literature contains several autonomous mechanisms for peer discovery using a social-unaware approach [2,3,4,5,6,7]. For example, the FlashLinQ protocol that was proposed in [2] uses frequency division multiplexing (FDM) to propagate the beacons through the network [2]. Notably, the beacons are transmitted both periodically and synchronously, and hence FlashLinQ provides an effective means of estimating the amount of consumed resources and timing the resource consumption to minimize resource snatching. The scheme that is presented in [3] replaces the FDM-based peer discovery process in [2], with a non-orthogonal multiple-access scheme that is referred to as Trellis Tone Modulation Multiple-Access (TTMMA). TTMMA uses single-tone transmissions and achieves long-distance discovery due to its low Peak-to-Average Power Ratio (PAPR). Furthermore, it makes a higher discovery capacity through its use of a non-orthogonal resource assignment mechanism possible. Based on the assumption of a synchronized superframe structure among the UEs, the peer discovery mechanism in [4] uses both a common channel and a group of channels to send beacons subject to certain rules and procedures that are designed to minimize the discovery time. To ensure compatibility with the standard LTE protocol, the UEs contained in [5] perform peer discovery by listening to the Sounding Reference Signal (SRS) channel, being originally designed for data uploading purposes in LTE networks and peer UEs can access it. However, the method is only capable of detecting the presence of peers, i.e., not active peers that are interested in D2D discovery and communications. Accordingly, the mechanism in [6] exploits the standardized uplink signal structure in the SRS channel to accomplish both the detection of the active peers and the identification of their beacons. Finally, in the peer discovery method that was proposed in [7], the UEs reside in one of five different states, namely, *Keep Alive*, *Advertise*, *Discover*, *Follow,* and *Passive*, depending on their behavior. The UEs in each state then employ a particular beacon rate that is chosen in advance in such a way as to maximize the power saving in the network.

The literature contains various proposals for network-assisted peer discovery methods [8,9,10,11,12]. In [8], the UEs advertise their presence using a random access mechanism and the BS accepts the D2D requests, allocates resource blocks (RB), and chooses an appropriate beacon rate for each UE, depending on the number of requests that they produce. In the peer discovery method that is proposed in [9], the UEs authorized to perform discovery in a given discovery interval contend to transmit their beacons in a time-frequency multiplexed pool of network-allocated resources. In [9], it is assumed that the transmitted beacons are always successful, i.e., other peers can successfully receive them. However, the method in [10] considers a more realistic network model that is based on the Poisson Point Process (PPP), in which the effect of the Channel State Information (CSI) on the performance of the D2D discovery process is taken into account by considering the interference that is imposed on the D2D pairs by nearby cellular users. In [11], each UE sends a preamble to the nearby UEs via a newly-introduced physical channel, and the UEs that receive this preamble send a corresponding report message to the BS by means of a normal random access procedure. The BS then allocates an uplink RB for each reported preamble, such that the UEs that initially sent the preamble can send a further report message to the BS. Finally, the BS, by comparing their reported preambles, identifies pairs of UEs that are in close proximity to one another. The method in [12] exploits the fact that some of the channel components of the UEs are spatially correlated to enable the BS to make a rough estimate of the UE locations by measuring these components. The BS then schedules the transmissions of the beacons that are sent by nearby UEs, such that nearby UEs transmit their beacons at similar times. 

Network-controlled methods have several important advantages over Autonomous and Network-Assisted methods, including low power consumption, reduced interference, and a low beacon transmission cost. Consequently, the authors in [13] proposed a peer discovery method based on the Angle of Arrival (AOA) measurements that were obtained by multiple BSs and further analyzed the performance of network-controlled D2D discovery in random spatial networks. The authors in [14] proposed a centralized novel approach, called ROOMMATEs, which utilizes the ubiquitous WiFi network, which combines with BS for indoor peer discovery. ROOMMATEs is an unsupervised approach that can provide different granularity location information. However, none of the studies in [2,3,4,5,6,7,8,9,10,11,12,13,14] consider the potential for improving the peer discovery performance by exploiting the social behaviors of the UEs in the network.

In fact, the literature contains very few proposals for social-aware peer discovery mechanisms [17,18,19,20,21]. Among those methods that have been proposed, three schemes [17,18,19] adopt an autonomous approach. The method in [17] focuses on the security of the data transmissions and it chooses UEs with high social ties (i.e., high trust) to perform D2D communications. However, by adopting such an approach, it is possible that no UEs may be available for pairing. Consequently, the authors in [18] proposed a two-hop pairing process, in which any UE failing to find a trusted UE with its wanted contents for D2D communications sends a request to all the one-hop neighbors of its trusted UEs. The method in [19] uses three key social attributes, namely the trust degree, the similarity degree between UEs, and the center degree of each UE, to construct a unified metric with which to construct neighbor lists for peer discovery. Reference [20] addresses the content delivery problem that is related to optimization of peer discovery and resource allocation by combining both the social and physical layer information in D2D networks. The social relationship, which is modeled as the probability of selecting similar contents, is used as a weight to characterize the impact of social features on D2D pair formation and content sharing. The peer discovery in the physical layer depends on the received signal power by UEs, while the social relationship is mainly used to optimize the context delivery. The method in [21], which is called Social-Based Grouping (SBG), adopts a network-assisted approach to perform peer discovery. UEs are grouped based on their social feature and centrality, and the UEs in the same group are assigned the same beacon rate. The BS, in accordance with the rate at which each UE contacts the other UEs in the network, determines the beacon rate. The UE with more contacts will send beacons at a higher rate.

Similar to SBG, our study also focuses on the network-assisted approach to perform peer discovery. However, between [21] and our study, there are some big differences: (1) SBG determines the beacon rate with only considering the social feature, while ERRD determines it with not only considering the social feature, but also the harvested power. (2) The UEs in SBG are grouped and the UEs in the same group are assigned the same beacon rate due to its high complexity, while each UE in ERRD has its individual beacon rate due to its low complexity. (3) Since ERRD extra considers the harvested power to determine more suitable beacon rate, its PDR can be significantly better than that of SBG. This can be easily observed in Section 5. 

## 3. Problem Description

This section commences by introducing the system model and associated notations. The problem statement is then formally defined.

### 3.1. System Model

The interval between the time *t* at which UE *i* comes within range of UE *j* and the time at which it was last within range of UE *j*, *t_0_*, is referred to as the D2D contact interval of the two UEs, *CI_i,j_*, and it is defined as

(1) $$CI_{i,j}=\underset{t}{\min}\left\{\left(t-t_{0}\right):\|L_{i}-L_{j}\|\leq R_{i,j},\;t>t_{0}\right\},$$

where ‖.‖ denotes the distance between the two UEs; *L_i_* and *L_j_* are the locations of UEs *i* and *j*, respectively; and, *R_i,j_* is the coverage range between the two UEs.

Based on the contact interval between the two UEs, the D2D contact rate between them, denoted as *λ_i,j_*, is defined as

(2) $$\lambda_{i,j}=\;\frac{1}{E\left[CI_{i,j}\right]},$$

where *E*[.] denotes the expectation. The average contact rate of UE *i*, denoted as *λ_i_*, is then computed as

(3) $$\lambda_{i}=\;\frac{\sum_{j=1,j\neq i}^{N}\lambda_{i,j}}{N-1},$$

where *N* is the total number of UEs in the network. Let $CI_{i,j}$ follow a cumulative distribution function (CDF) of $F_{CI_{i,j}}\left(x\right)$, with rate *λ_i,j_*. Assume further that $F_{CI_{i,j}}\left(x\right)$ is a uniform distribution, and can thus be written as

(4) $$F_{CI_{i,j}}\left(x\right)=\{\begin{array}{c} 0,\;\;\;\;\;\;\;\;x<0,\\ \frac{x}{2E\left[CI_{i,j}\right]},\;\;0<x<2E\left[CI_{i,j}\right],\\ \;1,\;\;x>2E\left[CI_{i,j}\right]. \end{array}$$

Let the social ratio of UE *i* be defined as the square root of its average contact rate over the sum of the square root of each average contact rate, i.e.,

(5) $$r_{i}=\frac{\lambda_{i}^{\frac{1}{2}}}{\sum_{j=1}^{N}\lambda_{j}^{\frac{1}{2}}}.$$

In modeling the energy-harvesting environment, it is assumed that the charging devices (CDs) and BS convert their power into RF signals. Furthermore, any UEs within the coverage of these CDs or the BS acquire this RF signal and then convert it into power through special equipment. The power that is obtained by UE *i* from energy harvesting, denoted as *OP_i_*, is thus calculated as

(6) $$OP_{i}=\sigma \left(CDP_{m}\|L_{i}-CDL_{m,\;i\in CDR_{m}}\|{}^{-\upsilon }\right),$$

where σ is an energy harvesting efficiency factor that reflects the ability of UE *i* to change the RF signal into power; *v* is the path loss exponent that is caused by interference, which increases with an increasing distance; and, *L_i_* is the location of UE *i*. In addition, *CDP_m_*, *CDR_m_*, and *CDL_m_* are the transmitted power, coverage, and location, respectively, of CD *m*, when UE *i* lies within its coverage [30,31,32].

### 3.2. Problem Statement

Before formally defining the problem statement, the used notations are listed in Table 2. As shown, the notations fall into six categories that relate to the system, range, power, location, contact, and beacon, respectively. The system parameters define the number of UEs in the network and the total peer discovery time, respectively, while the range parameters describe the coverage of the devices. The power parameters define the transmission powers of the devices and the various power variables that are used in the ERRD model. The location parameters define the positions of the devices. The contact parameters describe the contact behaviors of the UEs. Finally, the beacon parameters define the beacon rates of the UEs.

**Figure 1:**
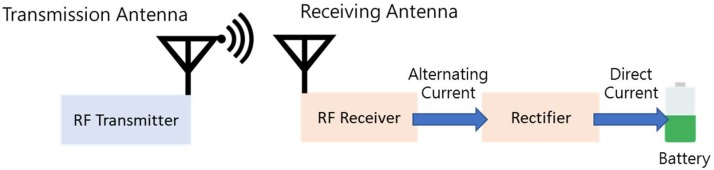
The energy harvesting technology considered in this study.

The energy harvesting technology that is considered in this study is wireless power transfer (WPT) via radio frequency (RF), as shown in Figure 1 [23]. The basic idea is that the RF transmitter transmits radio signal towards the receiving antenna at the desired frequency and power level. The RF receiver then applies electromagnetic radiation to charge the battery, that is, the receiving antenna receives the traveling signal and the rectifier converts the alternating current (AC) to direct current (DC) to charge the battery. The energy carrier can be located at 2.4 GHz or 5 GHz frequency band under the considerations that these bands are internationally reserved for Industrial, Scientific, and Medical (ISM) purposes. Energy harvesting can explore sufficient power sources in significant radio coverage by increasing the transmitting power under the regulation of the government.

This study attempts to determine the suitable beacon rates of all UEs to explore most peers in a D2D network under the constraints of budget power, the limitation of overall power consumption. This study further introduces a new scenario, *i.e.*, energy harvesting, which previous studies did not consider. The energy harvesting enables UEs within charging areas to procure additional power from surrounding energy sources. Thus, under considering the UE may be out-of-energy and the study determines the suitable beacon rates of all UEs to maximize PDR according to the budget power and harvested power.

The problem that we investigated is formally described, as follows. First, for a beacon rate of UE *i* equal to *μ_i_*, the peer discovery ratio (PDR) of the UE can be calculated as [33] 

(7) $$\mathrm{PDR}=1-2\mu_{i}^{2}\int_{0}^{\frac{1}{\mu_{i}}}\int_{0}^{y}F_{CI_{i,j}}\left(x\right)dxdy.$$

The second term denotes the missing probability and the probability UE *i* cannot detect the contact. In other words, it is the probability that the UE *i* cannot find other peers. 

According to the arrival contact rate of each UE, the aim of the peer discovery process is to maximize the total PDR, as

(8) $$\overset{N}{\underset{i=1}{\sum }}\lambda_{i}\left(1-2\mu_{i}^{2}\int_{0}^{\frac{1}{\mu_{i}}}\int_{0}^{y}F_{CI_{i,j}}\left(x\right)dxdy\right).\;s.t.\;\sum_{1}^{N}\mu_{i}\times TP\leq BP,$$

where *TP* is the transmission power used to send a beacon message and *BP* is the total budget power quantum of the network. However, if a UE is out-of-energy, it cannot find any peers in the network and any other peers cannot find it. Thus, in performing the peer discovery process, the objective given in Equation (8) should be modified, as follows:

(9) $$\max\;\mathrm{PDR}=\overset{N}{\underset{i=1}{\sum }}\{\begin{array}{r} \lambda_{i}\left(1-2\mu_{i}^{2}\int_{0}^{\frac{1}{\mu_{i}}}\int_{0}^{y}F_{CI_{i,j}}\left(x\right)dxdy\right),\;\;{\;P}_{i}>0\\ 0,\;\;P_{i}\leq 0 \end{array}\;s.t.\;\sum_{1}^{N}\mu_{i}\times TP\leq BP,$$

where *P_i_* is the power reserved for peer discovery of UE *i*. Differing from Equation (8), the UE cannot find other peers and it cannot be found by any other peers when $P_{i}\leq 0$ in Equation (9), because it is a more reasonable condition. Also note that the constraint denotes that the total power consumption must be less than the budget power quantum.

Thus, the problem statement is formally given, as follows.

**Input:** the parameters marked input in Table 2.

**Output:** the beacon rate of UE *i*, $\mu_{i},\;\forall i.$

**Objective:** $\max\;\mathrm{PDR}=\overset{N}{\underset{i=1}{\sum }}\{\begin{array}{r} \lambda_{i}\left(1-2\mu_{i}^{2}\int_{0}^{\frac{1}{\mu_{i}}}\int_{0}^{y}F_{CI_{i,j}}\left(x\right)dxdy\right),\;\;P_{i}>0\\ 0,\;\;P_{i}\leq 0. \end{array},$

**Constraint:**$\overset{N}{\underset{1}{\sum }}\mu_{i}\times TP\leq BP$.

## 4. Solutions

Before introducing the ERRD algorithm in detail, the overall operations of UEs and BS are first described. The peer discovery is conducted per time period *T*. At the beginning of time period, each UE will send its ID and its harvested power quantum to BS. Thus, BS will know which UEs in its coverage and immediately extracts their social features from the database. According to UEs’ social features and the harvested power quantum, and the pre-determined budget power quantum, BS can run ERRD to determine the beacon rate for each UE, and then sends the determined beacon rate to the corresponding UE. After receiving this rate, the UE will send the beacons accordingly. Since each UE only calculates the harvested power quantum, according to Equation (8), its operation is very simple. Therefore, below, we focus on describing the algorithm in BS, i.e., ERRD.

In D2D communications, the beacon rate is proportional to the amount of consumed power. Hence, the ERRD algorithm that is proposed in this study first virtually allocates a proper power quantum to each UE and then determines the corresponding beacon rate according to this allocated power quantum. As described in the following sub-sections, ERRD comprises three steps, namely *Social Ratio Allocation* (SRA), *Energy Ratio Allocation* (ERA), and *Beacon Rate Decision* (BRD). SRA first allocates the budget power among the UEs, depending on their social ratios. For each UE, ERA then adjusts this allocated power quantum according to the amount of energy that is harvested by the UE. Finally, BRD computes the beacon rate of each UE, depending on the adjusted allocated power quantum.

Below, the concept of ERRD is first explained. As ERRD is composed of three steps, we describe SRA, ERA, and BRD in sequence in Section 4.1. After describing the concept of ERRD, we formally exhibit the pseudo code of ERRD in Section 4.2. Finally, an example to illustrate the overall ERRD operation is given in Section 4.3.

### 4.1. ERRD Algorithm

#### 4.1.1. Social Ratio Allocation

Let *AP* be the total allocated power quantum and *GP_i_* be the amount of power that is allocated to UE *i*. Since the peer discovery process is subject to the constraint that the total consumed power must be less than or equal to budget power *BP*, *AP* is initialized as *BP*. According to [21], when $F_{CI_{i,j}}\left(x\right)$ has a uniform distribution (as shown in Equation (4)), the maximum PDR is achieved when the beacon rate of each UE is set proportional to its social ratio, i.e., $\mu_{i}\propto r_{i}.$ Thus, the initially allocated power quantum for every UE is given by $GP_{i}=AP\times r_{i}$.

#### 4.1.2. Energy Ratio Allocation

The ERA step is the most critical step in ERRD. In order to properly explain the step, it is appropriate to introduce the following intuitive thought regarding the approach for allocating the power quantum, depending on the amount of power that is obtained from energy harvesting. The smart approach employed by ERRD is then introduced.

- Intuitive thought

The SRA step in the ERRD algorithm allocates the budget power quantum among the UEs based on their social ratios. However, some UEs can obtain power from energy harvesting, and hence their obtained power quantum *OP_i_* may exceed the allocated power quantum *GP_i_*. In this case, the UEs can utilize the obtained power quantum, rather than the originally allocated power quantum to perform their beacon transmissions. The unused portion of the allocated power quantum can then be returned to *AP* for the re-allocation to other UEs. Conversely, if *OP_i_* is less than *GP_i_*, i.e., the UE only acquires little (or no) energy via harvesting, the UE uses the original allocated power quantum *GP_i_* and returns its obtained power quantum *OP_i_* to *AP*. Combining these two cases, the unused power quantum of UE *i* is given by the minimum of *OP_i_* and *GP_i_*, i.e., min(*OP_i_*, *GP_i_*).

Since *AP* and *GP_i_* are dependent*,* it is necessary to obtain them alternately while using an iterative approach. Let $x^{k}$ denote the value of *x* in the *k*-th iteration and *k* = 0 denote the initial value. Therefore, the initial value of *AP*, $AP^{0}$, is set as the budget power quantum, *BP*, and it is allocated to each UE in accordance with $r_{i}$, i.e., $P_{i}^{0}=AP^{0}\times r_{i}$. Any unused power quantum must be returned to *AP*. Consequently, *AP* is equal to the sum of *BP* and the returned power quantum of all the UEs, i.e.,

(10) $$AP_{}^{k+1}=BP+\overset{N}{\underset{i=1}{\sum }}\min\;(OP_{i},\;GP_{i}^{k}),\;k\geq 0.$$

Once $AP^{k+1}$ is calculated, the new allocated power quantum of each UE can be obtained as $GP_{i}^{k+1}=AP^{k+1}\times r_{i}$, and used to calculate$\;AP_{}^{k+2}$ accordingly. As shown in Lemma 1, *AP* is non-decreasing as the number of iterations increases.

**Lemma** **1:** $AP^{k+1}\geq AP^{k},\;\forall k\geq 0$.

**Proof.** 

1. As $AP^{0}=BP$ and $AP^{1}=BP+\overset{N}{\underset{i=1}{\sum }}\min(OP_{i}+GP_{i}^{0})$, it follows that $AP^{1}\geq AP^{0}$ since all $OP_{i}$ and $GP_{i}^{0}$ are non-negative. Thus, *k* = 1 holds.
2. Suppose that k=n−1 holds, i.e., $AP_{}^{n}\geq AP_{}^{n-1}.$ $AP_{}^{n}\geq AP_{}^{n-1}$ implies that $GP_{i}^{n}\geq GP_{i}^{n-1},\;\;1\leq i\leq N$ since $GP_{i}^{n}=AP^{n}\times r_{i}$. Hence,
  $$\begin{array}{c} BP+\overset{N}{\underset{i=1}{\sum }}\min(OP_{i},GP_{i}^{n})\geq BP+\overset{N}{\underset{i=1}{\sum }}\min(OP_{i},GP_{i}^{n-1})\\ \;\Rightarrow AP_{}^{n+1}\geq AP_{}^{n} \end{array}$$
  Thus, k=n holds.
3. From mathematical induction, $AP^{k+1}\geq AP^{k},\;\forall k\geq 0$.  □

Intuitively, $AP_{}^{k}$ can be iteratively calculated until no further change in its value is obtained, i.e., $AP_{}^{k+1}=AP_{}^{k}$. However, while such an approach is technically feasible, it requires many iterations to converge, causing high complexity, and it is hence impractical for real-world peer discovery applications. Consequently, the following smart approach is proposed instead.

- Smart approach

According to the relationship between *OP_i_* and *GP_i_*, ERRD classifies the UEs into two groups, namely *G*_1_ when $OP_{i}<GP_{i}$ and *G*_2_ when $OP_{i}\geq GP_{i}$. Therefore, Equation (10) can be re-formulated as

(11) $$AP^{k+1}=BP+\underset{i\in G_{1}^{k}}{\sum }OP_{i}+\underset{i\in G_{2}^{k}}{\sum }GP_{i}^{k}.$$

**Lemma** **2:** *In each iteration, the UEs in G*_2_*may be shifted to G*_1_*, but the UEs in G*_1_*cannot be shifted to G*_2_.

**Proof.** 

1. From Lemma 1, *AP* is non-decreasing and $GP_{i}$ is also non-decreasing, i.e.,$\;GP_{i}^{k+1}\geq GP_{i}^{k},\;\forall i.$
2. Since $OP_{i}$ is fixed, the condition $OP_{i}^{}<GP_{i}^{k}$ implies that $OP_{i}^{}<GP_{i}^{k+1}$. Therefore, any member in *G*_1_ will not be shifted to *G*_2_.
3. Consequently, in each iteration, only the UEs in *G*_2_ may be shifted to *G*_1_, but no UEs in *G*_1_ will be shifted to *G*_2_.  □

From Lemma 2, the ERA process seeks to shift any UEs belonging to *G*_2_ to *G*_1_ if possible in each iteration in order to reduce the convergence time. However, to achieve this, two issues must first be addressed, namely (1) which UE should be considered first to be shifted and (2) whether this UE can actually be shifted.

ERA determines the answer to the first issue by inspecting the ratio of the obtained power quantum over the corresponding social ratio, i.e., *OPR_i_* = $OP_{i}/r_{i}$. A smaller value of *OPR_i_* implies that *OP_i_* is more likely to be less than *GP_i_*. In other words, UE *i* is more likely to belong to *G*_1_. Thus, in the *k*-th iteration, according to the current grouping, UE *m^k^*, whose $OPR_{m^{k}}$ is the smallest among those of all the UEs in $G_{2}^{k}$, is chosen as the pivot UE, and it is most likely to be shifted from $G_{2}^{k}$ to $G_{1}^{k}$, as described in the following lemma.

**Lemma** **3:** *The pivot UE m^k^ among*$G_{2}^{k}$*is most likely be shifted to*$G_{1}^{k}$.

**Proof.** 

1. $OPR_{i}=\frac{OP_{i}}{r_{i}}\Rightarrow \frac{OPR_{i}}{AP_{}^{k}}=\frac{OP_{i}}{AP_{}^{k}\times r_{i}}\Rightarrow \frac{OPR_{i}}{AP_{}^{k}}=\frac{OP_{i}}{GP_{i}^{k}}\Rightarrow OPR_{i}=\frac{OP_{i}}{GP_{i}^{k}}AP_{}^{k}$.
2. In the *k*-th iteration, as $AP_{}^{k}\;$is fixed, UE *m^k^* has the smallest *OPR* in *G*_2_, i.e., $\frac{OP_{m^{k}}^{}}{GP_{m^{k}}^{k}}$ is the smallest, and hence $OP_{m^{k}}^{}$ is most likely to be less than $GP_{m^{k}}^{k}.$ Consequently, UE *m^k^* is most likely to be shifted from $G_{2}^{k}$ to $G_{1}^{k}$.  □

ERA considers whether or not this UE can actually be shifted after determining the pivot UE. To achieve this, a virtual critical point, defined as $OP_{m^{k}}=GP_{m^{k}}$, is considered. At this critical point, the virtual total allocated power quantum, $VAP^{k}$, obtained using $m^{k}$ as the baseline, is given as

(12) $$VAP^{k}=BP+\underset{i\in G_{1}^{k}}{\sum }OP_{i}+\underset{i\in G_{2}^{k}}{\sum }GP_{i}^{k}\;=BP+\underset{i\in G_{1}^{k}}{\sum }OP_{i}+\underset{i\in G_{2}^{k}}{\sum }GP_{m^{k}}\times \frac{r_{i}^{}}{r_{m^{k}}^{}}\;=BP+\underset{i\in G_{1}^{k}}{\sum }OP_{i}+\underset{i\in G_{2}^{k}}{\sum }OP_{m^{k}}\times \frac{r_{i}^{}}{r_{m^{k}}^{}}.$$

The virtual allocated power quantum for $m^{k}$ is then obtained as $VGP_{m^{k}}^{k}$=$VAP^{k}\times r_{m^{k}}$. The virtual critical point represents the threshold between $G_{2}^{k}$ and $G_{1}^{k}$. Thus, checking whether $VGP_{m^{k}}^{k}$ is more than$\;OP_{m^{k}}^{}$ provides an efficient means of determining whether UE $m^{k}$ belongs to $G_{2}^{k}$ or $G_{1}^{k}.$ If $VGP_{m^{k}}^{k}$ is larger than$\;OP_{m^{k}}^{}$, the pivot UE $m^{k}$ should be shifted from $G_{2}^{k}$ to $G_{1}^{k}$ and the iteration process should continue to the next round. Otherwise, the pivot UE $m^{k}$ should remain in $G_{2}^{k}$. As the pivot UE *m^k^* among $G_{2}^{k}$ is the most likely be shifted to $G_{1}^{k}$, the other UEs belonging to $G_{2}^{k}$ also remain in $G_{2}^{k}$. In other words, the grouping process is complete and no further changes in the memberships of *G_1_* and *G_2_* are required.

Once the grouping process is finished (in iteration *), $GP_{m^{*}}^{*}\;$can be computed as

(13) $$\frac{\;GP_{m^{*}}^{*}}{r_{m^{*}}^{}}=BP+\underset{i\in G_{1}^{*}}{\sum }OP_{i}+\underset{i\in G_{2}^{*}}{\sum }\;GP_{m^{*}}^{*}\times \frac{r_{i}}{r_{m^{*}}^{}}.$$

After solving Equation (13), $GP_{m^{*}}^{*}$ can be obtained as

(14) $$\;GP_{m^{*}}^{*}=\frac{r_{m^{*}}^{}\times \left(BP+\sum_{i\in G_{1}^{*}}OP_{i}\right)}{1-\sum_{i\in G_{2}^{*}}r_{i}}.$$

AP^*^ can then be computed as $AP^{*}=GP_{m^{*}}^{*}/r_{m^{*}}^{}$. In addition, each $GP_{i}^{*}$ can be computed with AP^*^. Finally, the allocated power quantum for each UE is set as the maximum between $GP_{i}^{*}$ and *OP_i_*.

#### 4.1.3. Beacon Rate Decision

In the BRD step, since *OP_i_* is considered, the sum of all the allocated power quanta is equal to the budget power quantum plus all the obtained power quanta, as shown in Lemma 4.

**Lemma** **4:** $\overset{N}{\underset{i=1}{\sum }}\underset{}{\max}\left(OP_{i},GP_{i}^{*}\right)=BP+\overset{N}{\underset{i=1}{\sum }}OP_{i}$.

**Proof.** 

1. For UE *i* in *G*_2_, the allocated power quantum is $GP_{i}^{*}$. As the UE has an obtained power quantum of $OP_{i}$, the allocated power quantum occupying the budget power quantum is equal to $GP_{i}^{*}-OP_{i}$. Thus, it follows that $\underset{i\in G_{2}^{*}}{\sum }\left(GP_{i}^{*}-OP_{i}\right)=BP$.
2. $\begin{array}{l} \overset{N}{\underset{i=1}{\sum }}\underset{}{\max}\left(OP_{i},GP_{i}^{*}\right)=\underset{i\in G_{1}^{*}}{\sum }OP_{i}+\underset{i\in G_{2}^{*}}{\sum }GP_{i}^{*}\\ \;=\underset{i\in G_{1}^{*}}{\sum }OP_{i}+\underset{i\in G_{2}^{*}}{\sum }\left(GP_{i}^{*}-OP_{i}\right)+\underset{i\in G_{2}^{*}}{\sum }OP_{i}\\ \;=\sum_{i=1}^{N}OP_{i}+\underset{i\in G_{2}^{*}}{\sum }\left(GP_{i}^{*}-OP_{i}\right)=BP+\sum_{i=1}^{N}OP_{i}\; \end{array}$.  □

Due to this value exceeding the *BP* quantum, the final allocated power quantum should be normalized through multiplication by *R*, i.e., the ratio of *BP* over the sum of the allocated power quanta. That is,

(15) $$R=\frac{BP}{\sum_{i=1}^{N}\underset{}{\max}\left(OP_{i},GP_{i}^{*}\right)}=\frac{BP}{BP+\sum_{i=1}^{N}OP_{i}}.$$

Finally, the *μ_i_* of each UE *i* is computed as the allocated power quantum multiplied by *R* and divided by *TP*.

### 4.2. Pseudo Code

Algorithm 1 shows the pseudo code of the ERRD algorithm, where lines 1–7 correspond to the SRA step, lines 8–24 describe the ERA step, and lines 25–29 are the BRD step. In the SRA step, lines 1–7 initialize the variables and compute *r_i_*, $GP_{i}$, and *OPR_i_* for all the UEs. In the ERA step, lines 8–12 classify the UEs into two groups. In particular, UEs with an *OP_i_* greater than or equal to *GP_i_* are shifted into group *G*_2_. Lines 15–17 then select UE *m* whose *OPR_m_* is the smallest among those of all the UEs in *G*_2_ as the pivot UE and compute *VAP* and *VGP_m_* accordingly. Line 18 checks whether the pivot UE meets the condition that *OP_m_* is larger than *VGP_m_*. If the condition holds, the final *AP* is computed in lines 19 and 20. Otherwise, lines 14–23 are repeated iteratively until this condition is reached. Finally, in the BRD step, *R* is calculated in line 25. Line 27 then computes *GP_i_* based on the final *AP* obtained in the ERA process and line 28 normalizes the allocated power quantum and computes the normalized *μ_i_*.

**Algorithm 1** Energy Ratio Rate Allocation Algorithm
$1:AP\leftarrow BP,\;G_{1}\leftarrow \left\{1\sim N\right\},\;G_{2}\leftarrow \emptyset$

$2:{\;\mathrm{Calculate}\;\mathrm{all}\;\lambda }_{i}\;\mathrm{from}\;CI_{i,j};\;\lambda_{ALL}\leftarrow \overset{N}{\underset{i=1}{\sum }}\lambda_{i}^{\frac{1}{2}}$

3:for i=1 to N do

$4:r_{i}\leftarrow \frac{\lambda^{\frac{1}{2}}}{\lambda_{ALL}}$

$5:GP_{i}\leftarrow AP\times r_{i}$

$6:OPR_{i}\leftarrow \frac{OP_{i}}{r_{i}}$

7:end for

8:   for i=1 to N do

$9:\;\;\;\;\;if\;OP_{i}\geq GP_{i}\;then$

$10:\;\;\;\;\;G_{2}\leftarrow G_{2}\cup^{\;}i,\;G_{1}\leftarrow G_{1}-i$

11:end if

12: end for

$13:\;if\;G_{2}\neq \emptyset \;then$

14:   repeat

$15:m\leftarrow \mathrm{argmin}\;OPR_{i},\;\forall i\in G_{2}$

$\begin{array}{ll} 16:VAP\leftarrow BP & +\left(\underset{i\in G_{1}}{\sum }OP_{i}\right)\\ & +\left(\underset{i\in G_{2}}{\sum }OP_{m}\times \frac{r_{i}}{r_{m}}\right) \end{array}$

$17:VGP_{m}\leftarrow VAP\times r_{m}$

$18:if\;OP_{m}\geq VGP_{m}\;then$

$19:\;GP_{m}\leftarrow \frac{r_{m}\times \left(BP+\sum_{i\in G_{1}}OP_{i}\right)}{1-\sum_{i\in G_{2}}r_{i}}$

$20:\;AP\leftarrow \frac{GP_{m}}{r_{m}}$

21:end if

$22:G_{1}\leftarrow G_{1}\cup^{\;}m,\;G_{2}\leftarrow G_{2}-m$

$23:until\;OP_{m}\geq VGP_{m}\;or\;G_{2}=\emptyset$

24: end if

$25:\;R\leftarrow \frac{BP}{BP+\sum_{i=1}^{N}OP_{i}}$

26: for i=1 to N do

$27:GP_{i}\leftarrow AP\times r_{i}$

$28:\mu_{i}\leftarrow \max\left(OP_{i},\;GP_{i}\right)\times \frac{R}{TP}$

29: end for


Note that the pseudo code of ERRD is run in BS at the beginning of each time period *T*. All allocating, returning, and re-allocating power quanta from the BS to the UEs are virtually calculated in BS, rather than the real transfer between BS and UEs. Therefore, the communication between BS and UEs happens when each UE sends its ID and its harvested power quantum to BS at the beginning of each time period, and BS sends the determined beacon rate to the corresponding UE after it has executed ERRD.

The time complexity of ERRD is calculated, as follows. The lines 1–2, lines 3–7, lines 8–12, line 25, and lines 26–28 individually requires O(*N*). The repeat loop of lines 14–23 are executed as most *N* times, because at least a UE will be shifted from *G*_2_ into *G*_1_ in each iteration. The time complexities of calculating *m* in line 15, *VAP* in line 16, and *GP_m_* in line 19 are O(*N*). Thus, the time complexity of the repeat loop is O(*N*^2^). Therefore, ERRD has low time complexity O(*N*^2^) and it can be implemented in a real-time environment.

### 4.3. Illustrative Example

The following discussions present an illustrative example to demonstrate the detailed operational steps of *ERRD*. It is assumed that the network contains five UEs with average social contacts, λ*_i_*, of 1, 4, 9, 16, and 25, respectively. It is further assumed that the *OP_i_* values of the five UEs are 5, 4, 5, 3, and 1, respectively. Finally, the total *BP* is assumed to be 15 and *TP* is set as 1.

The SRA step first computes *r_i_* in accordance with the average social contacts, λ*_i_*, i.e., $r_{1}=\frac{\sqrt{1}}{\sqrt{1}+\sqrt{4}+\sqrt{9}+\sqrt{16}+\sqrt{25}}=\frac{1}{15}$. Thus, $r_{1}$, $r_{2}$, $r_{3}$, $r_{4}$, and $r_{5}$ are obtained as $\frac{1}{15},\;\frac{2}{15}.\;\frac{3}{15},\;\frac{4}{15}$, and$\;\frac{5}{15}$, respectively (see Table 3). For each UE, $GP_{i}$ is then computed as the product of *AP*, which is initialized as *BP*, and $r_{i}$, i.e., $GP_{i}=AP\times r_{i}$. In other words, $GP_{1}$, $GP_{2}$, $GP_{3}$, $GP_{4}$ and $GP_{5}$ are obtained as 1, 2, 3, 4, and 5, respectively. In addition, *OPR_i_* is calculated as $OP_{i}/r_{i}$. For example, *OPR*_1_=$\frac{5}{\frac{1}{15}}=75$. The UEs are then classified into two groups, namely *G*_1_ or *G*_2_, by comparing $OP_{i}$ with $GP_{i}$. In the present example, $OP_{1}$, $OP_{2}$, and $OP_{3}$ are greater than $GP_{1}$, $GP_{2}$, and $GP_{3}$, respectively, while $OP_{4}$ and $OP_{5}$ are less than $GP_{4}$ and $GP_{5}$, respectively. Consequently, group *G*_1_ is determined to be {4, 5}, while group *G*_2_ is obtained as {1, 2, 3}.

Based on the results that are presented in Table 3, the ERA step selects UE 3 as the pivot UE, since the value of *OPR*_3_ is the smallest among all of the *OPR* values in *G*_2,_ To check whether the grouping process is finished, ERA uses the ratio of *r_i_* over *r*_3_ to compute *VAP* as $BP+OP_{5}+OP_{4}+OP_{3}\times \frac{r_{3}}{r_{3}}+OP_{3}\times \frac{r_{2}}{r_{3}}+OP_{3}\times \frac{r_{1}}{r_{3}}=15+3+1+\frac{5}{3}+\frac{10}{3}+5=29$. The *VAP* result is then used to compute *VGP*_3_ as $29\times \frac{3}{15}=\frac{29}{5}$_._ *OP*_3_ is less than *VGP*_3_, that is, the virtually allocated power quantum exceeds the obtained power quantum. Hence, UE 3 is shifted from group *G*_2_ to group *G*_1_.

The procedure that is described above is iteratively repeated until the grouping process is complete. For the present example, $G_{1}^{*}$ is obtained as {3, 4, 5} and $G_{2}^{*}$, as {1, 2}. Once the grouping process is finished, $GP_{2}^{*}$ is $GP_{m}^{*}\;$and in accordance with Equation (14) (line 19 in the algorithm), $GP_{2}^{*}$ is computed as $\frac{\frac{2}{15}\times \left(15+5+3+1\right)}{1-\left(\frac{1}{15}+\frac{2}{15}\right)}=$ 4. Thus, $AP_{}^{*}$ is obtained as $\frac{4}{\frac{2}{15}}=$30 (line 20). Finally, the values of $GP_{i}^{*}$ are computed using the determined value of $AP_{}^{*}$. Choosing the larger value between *OP_i_* and $GP_{i}^{*}$ for each UE, the total consumed power quantum is obtained as 5 + 4 + 6 + 8 + 10 = 33, which is equal to the sum of *BP* and all *OP_i_* (15+5+4+5+3+1). As the total consumed power quantum is larger than *BP*, normalization by $R=\frac{15}{33}=\frac{5}{11}$ is performed and used to compute *μ_i_*. For example, $\mu_{1}=5\times \frac{5}{11}\times \frac{1}{TP}=\frac{25}{11}$. Table 4 shows the final results for all the UEs.

## 5. Performance Evaluation

The PDR performance of the proposed ERRD algorithm was compared with that of the previously reported Social-Based Grouping (SBG) algorithm [21]. In SBG, the UEs are grouped based on their social feature: centrality and the UEs in the same group are assigned the same beacon rate. In the present simulations, SBG classifies the UEs into three groups.

### 5.1. Dataset and Environment

**Figure 2:**
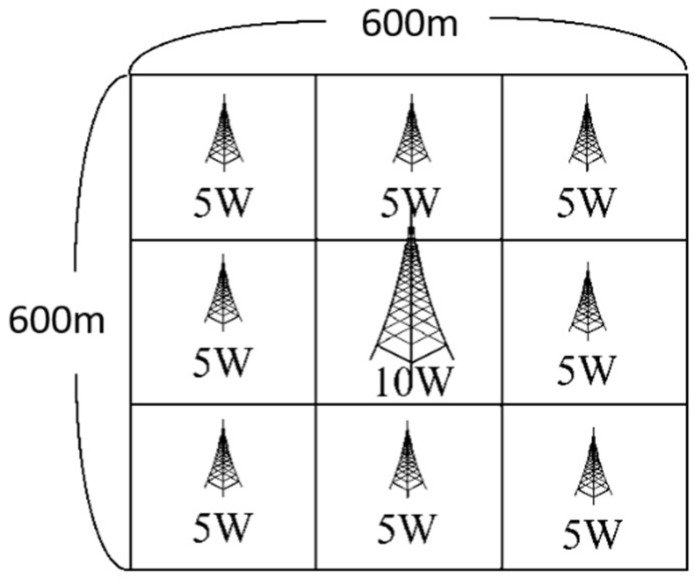
Simulation environment.

The simulations were performed using the *Infocom06* user mobility trace [34], which consisted of the D2D communication contacts of 98 individuals that were recorded over the *IEEE Infocom Conference* in 2006. The first half of the dataset was used to calculate the beacon rates from the contact rates of the UEs, while the second half was used to evaluate and compare the performance of the two schemes (ERRD and SBG). The dataset contains no information regarding the actual physical locations of the users and the BS. Consequently, in performing the simulations, an artificial environment for energy harvesting was created, with dimensions of 600 × 600 m^2^. The simulation field was partitioned into a 3 × 3 grid containing a BS with a 10-watt power in the center and eight CDs with a five-watt power distributed around the outside (see Figure 2). The UEs were uniformly deployed in the 3 × 3 grid initially and their locations were then randomly moved as the simulations proceeded to simulate the mobility of UEs. Thus, the UEs obtained harvested power from different CDs (or the BS) at different points in the simulation process.

The adopted dataset is the same as the dataset used in [21], because it is the most popular realistic dataset used in D2D communications, although it does not include the actual locations of UEs. To the best of our knowledge, there is no typical dataset that includes UE contacts and UE locations. Thus, in this study, we adopted the dataset in [21] and simulated the UE locations and their mobility by ourselves.

Each simulation was run for 1000 seconds. The plotted simulation results were then computed as the average value obtained over 100 simulations that were performed under identical conditions. The default environmental parameters were set as shown in Table 5. In accordance with the *Infocom06* dataset, the number of UEs was set as *N* = 98. Moreover, the path loss exponent, *v*, of the BS and CD RF transmissions was set as 2, the energy harvesting efficiency factor, σ, was set as 0.7, and the transmission power of the beacon messages was set as 20 mW. The *BP* of the peer discovery process (with a duration of 1000 s) was set as 10 W. Finally, the mobility (moving speed) of the UEs was set as 1 under the assumption that the mobility in the *Infocom06* dataset is 1. In general, as the moving speed of the UEs increases, the contact interval between them reduces, and *vice versa*. Thus, for a mobility value that is equal to 2, the contact interval between the UEs is equal to half that in the *Infocom06* dataset.

Some assumptions are made for simplifying the simulations: (1) The harvested power quantum is according to Equation (6), although this quantum will be affected by interferences or other factors in a real environment. (2) The locations of UEs are limited in this area 600 × 600 m^2^. That is, if a UE moves outside this area, then its location will be randomly located within this area. (3) The beacon is perfectly transmitted, i.e., it will not encounter any collision or be interfered by other noises. 

The simulations compared the performance of the ERRD algorithm with that of SBG under different settings of the *BP*, *CDP,* and *mobility* parameters. We compared ERRD and SBG, rather than other solutions because of two points. (1) As described in Section 2, ERRD and SBG belong to the same type: social-aware and network-assisted, but other solutions belong to different types. (2) As SBG used social features to adjust the beacon rate, it always outperforms other solutions without social-awareness. The evidence was exhibited in [21]. The UEs were classified into two types to facilitate the comparison between the two schemes, namely those with an increased beacon rate (IBR) and those with a decreased beacon rate (DBR), respectively. In the former case, the UEs using ERRD had a higher beacon rate than those using SBG, while, in the latter case, the beacon rate of the UEs using ERRD was lower than that of those using SBG.

### 5.2. Effect of BP

**Figure 3:**
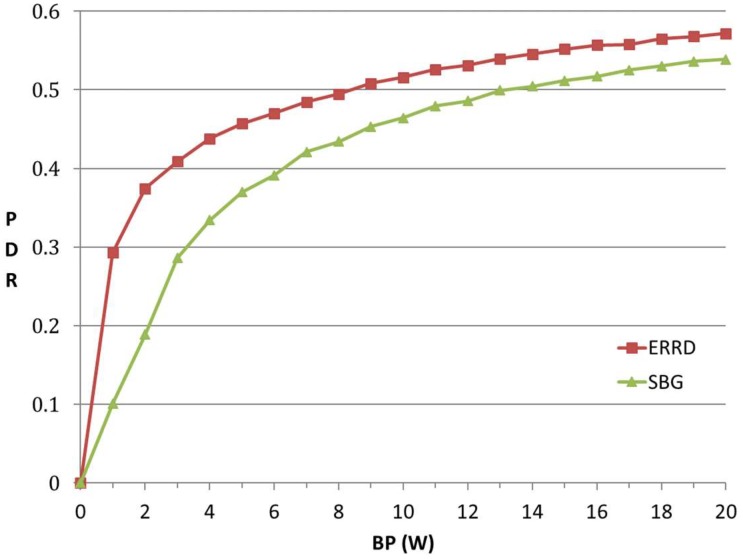
Peer Discovery Ratio (PDR) vs. total budget power quantum of the network (BP).

Figure 3 shows the PDR that was obtained under the two schemes for various values of the *BP* in the range of 0–20 W. For both schemes, the PDR exhibits a logarithmic-type increase as *BP* increases. This result is reasonable, since, intuitively, as the *BP* initially increases, the beacon rates of the UEs also increase, and hence a greater number of peers can be found. However, as the BP continues to increase, the number of originally unfound peers reduces, and hence the improvement in the PDR also reduces. The contact intervals in the *Infocom06* dataset are not uniformly distributed and some of the intervals are extremely short. Thus, if the beacon rate is assigned a very high value in an attempt to increase the number of discovered peers, the consumed *BP* significantly increases. Therefore, in practical implementations, the *BP* should be set in such a way as to achieve a satisfactory tradeoff between the PDR and the consumed *BP*.

For a given value of the *BP*, ERRD consistently achieves a higher PDR than SBG. For example, the PDR achieved under ERRD for a *BP* of 1 W is around 190% higher than that obtained under SBG, while for a *BP* of 20 W, the PDR is approximately 8% higher. This performance improvement can be attributed to two main factors. First, the IBR UEs using ERRD can send more beacons than those using SBG, and hence achieve a higher PDR. Second, although the DBR UEs using ERRD send fewer beacons than those using SBG, they have a longer lifetime. Consequently, the UEs can still achieve a higher PDR than those using SBG. However, the performance improvement that is offered by ERRD reduces with an increasing BP. This finding is reasonable, since, under a large *BP*, the amount of harvested energy is relatively smaller than that directly allocated by the BS. In other words, for most UEs, *OP_i_* < *GP_i_*, and hence the value of *GP_i_* under ERRD is similar to that under SBG. Consequently, the PDR performance gap between the two schemes reduces.

Some intermediate results can be observed to better understand the differences between ERRD and SBG. First, note that the overall power consumptions for ERRD and SBG are the same as the overall power consumption is always limited by the budget power quantum; so, showing this value is unnecessary. However, the power consumption of each UE is quite different for these two approaches. Thus, we observe two intermediate results: Number of out-of-energy UEs (NoE) and the coefficient of variation (CV) of power in UEs. The NoE represents the number of UEs that power quantum reserved for peer discovery has been exhausted, that is, the power consumption exceeds the value of the power quantum reserved for peer discovery plus the harvested power quantum. The out-of-energy UEs cannot find other peers and cannot be found by other peers. The CV represents the power distribution among the UEs. The lower CV means a better balance of UE battery power.

**Figure 4:**
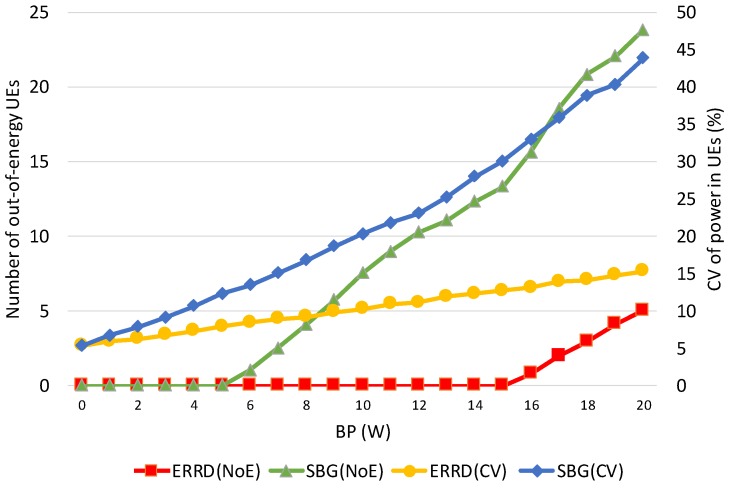
Number of out-of-energy User Equipments (NoE) and coefficient of variation (CV) vs. BP.

Figure 4 shows the NoE and CV that were obtained under the two schemes for various values of the *BP*. Observing this figure, NoE increases as *BP* exceeds a threshold. When BP increases, the UE can send more beacons. Therefore, the probability that a UE exhausts its battery power becomes larger, resulting in the increase of NoE. The out-of-energy UE appears when *BP* is 6 for SBG, while the out-of-energy UE appears when *BP* is 16 for ERRD. This is because SBG only considers the social ratio to send the beacons, while ERRD not only considers the social ratio, but also harvested power. When a UE has low harvested power and a high social ratio, ERRD reduces its beacon rate to reduce the probability of out-of-energy. However, in this case, SBG still lets this UE send beacons at a high rate, so it is very likely to be out-of-energy. On the other hand, CVs for ERRD and SBG almost linearly increase as *BP* increases, but SBG has a sharper slope. SBG generates many out-of-energy UEs and many UEs having much power, as they obtain much harvested power and send few beacons, resulting in more unbalanced battery power distribution among UEs, i.e., a higher CV. 

### 5.3. Effect of CDP

**Figure 5:**
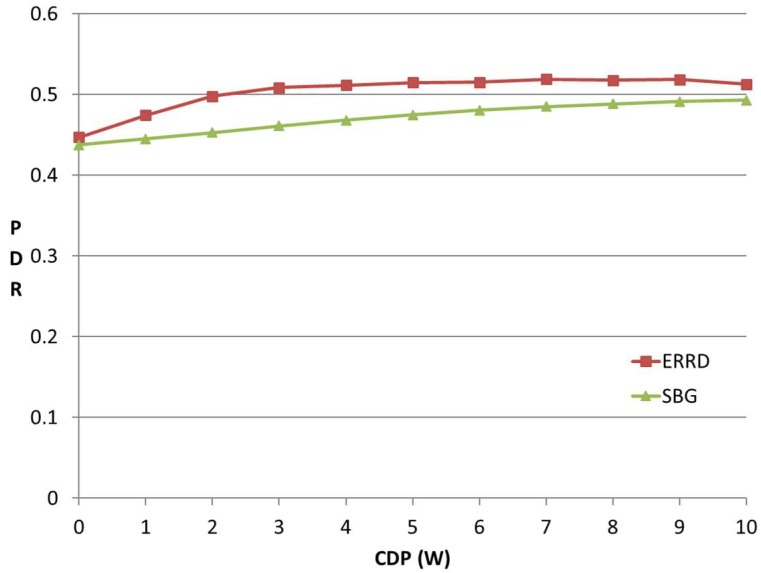
PDR vs. CDP.

Figure 5 shows the variation of the PDR with the *CDP*. In general, a larger *CDP* indicates that more UEs can harvest energy, or individual UEs can acquire a greater amount of energy. However, the total consumed power in the network is limited to *BP*, irrespective of the value assigned to *CDP*. In other words, for a larger *CDP*, ERRD cannot consume more power than *BP*, but can only allocate the power quantum to each UE more precisely, such that all of the UEs can more efficiently send their beacons. As shown in Figure 5, the PDR obtained under ERRD rapidly increases as the *CDP* first rises since the beacon rate decision made by ERRD reduces the probability that the UEs run out of energy. For a *CDP* value greater than 2, almost none of the UEs are out of energy. In this case, the PDR slightly increases, since the IBR UEs can find a greater number of peers. However, the PDR is not guaranteed to continuously increase when the number of UEs with an energy-harvesting capability exceeds a certain threshold. Therefore, in implementing the ERRD algorithm, a threshold should be set, whereby when the number of UEs with an energy-harvesting capability is greater than this threshold, ERRD should let some of the UEs store the harvested energy in their batteries, rather than expending it on beacon transmissions in order to provide power for other applications.

For the SBG scheme, the PDR also increases with an increasing *CDP*. However, it increases at a slower rate than under ERRD, since, even though SBG does not explicitly consider the power obtained from energy harvesting, the number of out-of-energy UEs still decreases as the number of UEs having an energy-harvesting capability increases.

**Figure 6:**
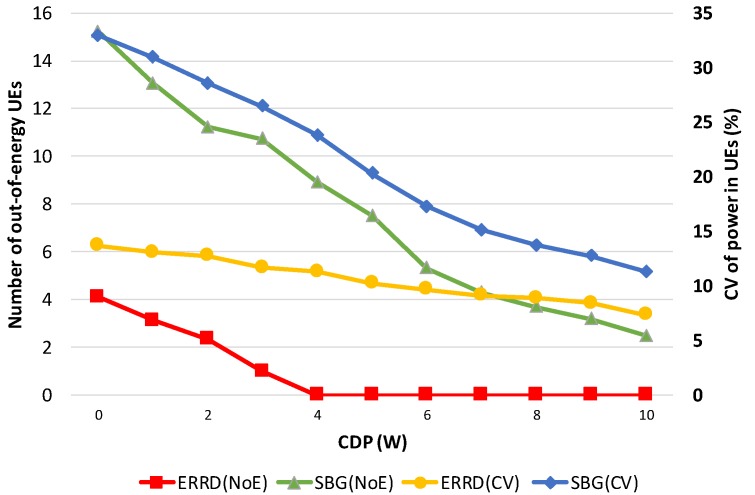
NoE and CV vs. CDP.

Figure 6 shows the NoE and CV that were obtained under the two schemes for various values of the *CDP*. Observing this figure, NoE decreases as *CDP* increases, because UEs can harvest more power. Therefore, the probability that a UE exhausts its battery power becomes lesser, resulting in the decrease of NoE. However, the NoE of SBG is significantly larger than that of ERRD, because of the reasons that are described in Figure 4. On the other hand, CVs for ERRD and SBG almost linearly decrease as *CDP* increases. When *CDP* is small, the UE in the BS coverage still can harvest much power, but the UEs in the CD coverage only harvest less power, resulting in a larger CV. Similar to Figure 4, we can see SBG has a sharper slope of CV than ERRD. 

### 5.4. Effect of Mobility

**Figure 7:**
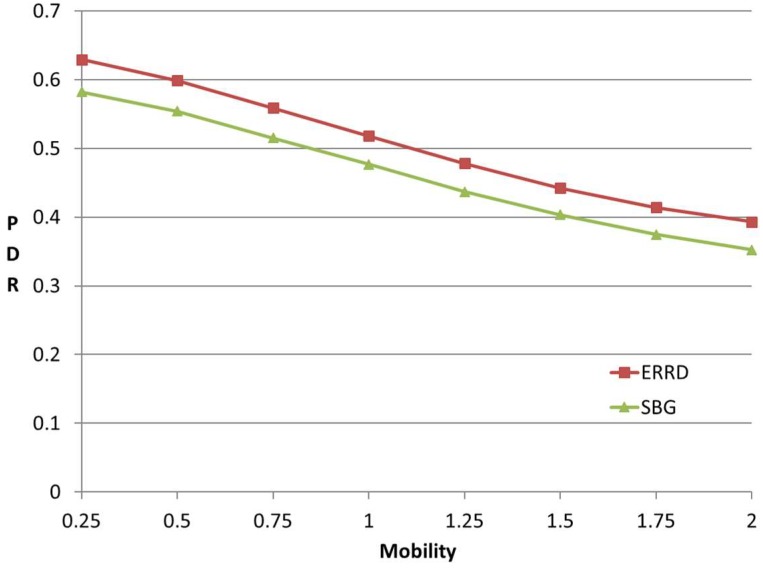
PDR vs. mobility.

Figure 7 shows the effect of the UE mobility on the PDR under the two schemes. As expected, the PDR reduces with an increasing mobility for both schemes, since, as the UEs move more rapidly, the contact rate between them increases, and hence the peers are less easily found for a given beacon rate. Nonetheless, the ERRD algorithm consistently outperforms SBG by around 10% for all the values of the UE mobility. Although the duration available for energy harvesting from one particular CD reduces as the UE mobility increases, the chance of harvesting energy from the other CDs increases. As a result, the UE mobility has no significant effect on the amount of harvested energy under the ERRD scheme. A similar tendency also occurs under the SBG scheme. Consequently, the performance advantage of ERRD over SBG is maintained, irrespective of the value of the UE mobility.

## 6. Conclusions and Future Works

This study has proposed a social-aware peer discovery mechanism, designated as ERRD, for energy harvesting-based D2D communications. ERRD first allocates the budget power of the network among the UEs, depending on their social ratios and then adjusts the allocated power quantum of each UE, depending on its harvested energy. Finally, ERRD sets the beacon rates of the UEs based on their adjusted power quanta subject to the constraint that the total power allocated to the UEs may not exceed the *BP* quantum of the network. ERRD improves the PDR performance by increasing the beacon rates of the high-energy-harvesting UEs, thereby increasing the number of peers that they can discover; and, reducing the beacon rates of the low-energy-harvesting UEs, thereby extending their lifetimes and prolonging the period for which they can participate in the peer discovery process. The simulation results have shown that ERRD outperforms the SBG scheme that is reported in the literature by around 8–190%, depending on the *BP* quantum of the network. The PDR performance of ERRD does not significantly increase as the *BP* increases beyond 10 W or the *CDP* exceeds 2. However, in mobile environments, ERRD retains a 10% performance advantage over SBG, irrespective of the moving speed of the UEs.

Currently, 5G D2D communications and energy-harvesting devices are not so popular to give a realistic example of use case. Therefore, the paper is more research-oriented, rather than system-oriented. However, we believe that D2D communications and energy-harvesting devices will become more popular in the future. The scenario that is considered in the study will actually happen and our proposed solution, ERRD, can be applied.

A complete D2D communication not only includes peer discovery, but also mode selection and resource allocation. Future studies will aim to establish a more comprehensive energy harvesting-based D2D communication model that includes mode selection and resource allocation. In its current form, ERRD considers the past behavior (sociality) and present condition (energy harvesting) of the UEs, but it does not consider the impact of the surrounding information, such as the number of peers in the network or the interference. Therefore, in future studies, ERRD will be extended to take such information into account in order to obtain a more robust estimation of the most appropriate beacon rate for D2D communications. Finally, the performance of ERRD in a realistic environment should be further investigated, as our study proves the outperformance of ERRD by simulations. For example, a UE who has a social-network account and, at the same time, can harvest energy can enhance how much of PDR. To observe this, ERRD should be realistically implemented in BS. We will pay the efforts on this implementation in the future.

## Figures and Tables

**Table 1:** Comparison of related works.

Category	Ref.	Type	Main Goal	Approach
Social-unaware	[2]	Autonomous	Min resource	UEs periodically and synchronously send beacons using FDM.
	[3]	Autonomous	Min resource	UEs use trellis tone modulation multiple-access scheme.
	[4]	Autonomous	Quick recovery	UEs use a common channel and a group of channels to send beacons.
	[5]	Autonomous	LTE compatible	UEs listen to SRS channel to identify nearby UEs.
	[6]	Autonomous	LTE compatible	UEs listen to SRS channel to detect active UEs.
	[7]	Autonomous	Min energy	UEs determine the beacon rate based on their state.
	[8]	Network-Assisted	Min collision	BS determines the beacon rate for UEs depending on the number of requests sent by UE.
	[9]	Network-Assisted	Min collision	UEs authorized to perform discovery contend to transmit beacons.
	[10]	Network-Assisted	Min interference	Similar to [9], but interference from cellular UEs imposed on D2D pairs is also considered.
	[11]	Network-Assisted	Min resource	UEs send preamble to nearby UEs and BS allocates uplink RBs for UEs.
	[12]	Network-Assisted	Beacon schedule	BS roughly estimates the location of UEs by measuring channel components.
	[13]	Network-Controlled	Min resource	BSs locate UEs by AOA.
	[14]	Network-Controlled	Min energy	Wifi scans are first used to determine the UE locations and BS then sends D2D broadcast.
Social-aware	[17]	Autonomous	Trust	UEs find trusted UEs.
	[18]	Autonomous	Two-hop pairing	UEs send requests to trusted UEs, which forward request to all one-hop UEs.
	[19]	Autonomous	Hybrid attributes	UEs use three key social attributes to construct neighbor lists.
	[20]	Autonomous	Max content delivery	Social relationship is used as a weight for D2D pair formation and content sharing.
	[21]	Network-Assisted	Max PDR	BS determines the beacon rate based on contact rate.
	ERRD	Network-Assisted	Max PDR	BS adjusts beacon rate based on harvested energy amount.

FDM: Frequency Division Multiplexing, SRS: Sounding Reference Signal, AOA: Angle of Arrival.

**Table 2:** Notation table.

Category	Notation	Description	Property
System	*N*	Number of UEs	Input
	*T*	Total time of peer discovery	Input
Range	$R_{i,j}$	Converge between UE *i* and *j*	Input
	$CDR_{m}$	Coverage of CD *m* or BS (*m* = 0 represents BS)	Input
Power	*BP*	Budget power	Input
	*TP*	Consumed power in sending a beacon	Input
	$CDP_{m}$	Transmission power of CD *m* or BS (*m* = 0 represents BS)	Input
	*σ* ∈ (*0,1*]	Energy harvesting efficiency factor	Input
	*v*	Path loss exponent	Input
	$P_{i}$	Owned power of UE *i*	Input
	$OP_{i}$	Obtained power of UE *i*	Variable
	$GP_{i}$	Allocated power of UE *i*	Variable
	*AP*	Total power allocated to UEs	Variable
Location	$L_{i}$	Location of UE *i*	Input
	$CDL_{m}$	Location of CD *m* or BS (*m* = 0 represents BS)	Input
Contact	$CI_{i,j}$	Contact interval of UEs *i* and *j*	Input
	$\lambda_{i,j}$	Contact rate of UEs *i* and *j*	Variable
	$\lambda_{i}$	Average contact rate of UE *i*	Variable
	$r_{i}$	Social ratio of UE *i*	Variable
	$OPR_{i}$	Ratio of *OP_i_* over *r_i_*	Variable
Beacon	$\mu_{i}$	Beacon rate of UE *i*	Output

**Table 3:** Initialized values.

UE ID	*r_i_*	*OP_i_*	*GP_i_*	*OPR_i_*	Set
1	$\frac{1}{15}$	5	1	75	*G*_2_
2	$\frac{2}{15}$	4	2	30	*G*_2_
3	$\frac{3}{15}$	5	3	25	*G*_2_
4	$\frac{4}{15}$	3	4	$\frac{45}{4}$	*G*_1_
5	$\frac{5}{15}$	1	5	3	*G*_1_

**Table 4:** Final results of the illustrative example.

UE ID	*r_i_*	*OP_i_*	*GP_i_*	Maximum	*μ_i_*
1	$\frac{1}{15}$	5	2	*OP_i_*: 5	$\frac{25}{11}$
2	$\frac{2}{15}$	4	4	*OP_i_*: 4	$\frac{20}{11}$
3	$\frac{3}{15}$	5	6	*GP_i_*: 6	$\frac{30}{11}$
4	$\frac{4}{15}$	3	8	*GP_i_*: 8	$\frac{40}{11}$
5	$\frac{5}{15}$	1	10	*GP_i_*: 10	$\frac{50}{11}$

**Table 5:** Default parameter settings in performance evaluation simulations.

Parameter	Default Value
*N*	98
*T*	1000 seconds
*v*	2
σ	0.7
*CDP_m_*	10 watts (m = 0, i.e., BS) 5 watts (m = 1–8)
*CDR_m_*	100 meters
*BP*	10 watts
*TP*	20 milliwatts
*P_i_*	300 milliwatts
Mobility	1
